# Effects of Different Irrigation Regimes on Root Growth and Physiological Characteristics of Mulch-Free Cotton in Southern Xinjiang

**DOI:** 10.3390/life15030435

**Published:** 2025-03-10

**Authors:** Feiyan Su, Ziyang Guo, Bingrong Wu, Jichuan Wang, Shuangrong Chen

**Affiliations:** 1 Agricultural College, Tarim University, Alar 843300, China; s10757232007@outlook.com (F.S.); guoziyang199911@outlook.com (Z.G.); 10757223012@stumail.taru.edu.cn (B.W.); csr10757241007@outlook.com (S.C.); 2 Key Laboratory of Genetic Improvement and Efficient Production for Specialty Crop in Arid Southern Xinjiang of Xinjiang Corps, Alar 843300, China

**Keywords:** Southern Xinjiang, mulch-free cotton, irrigation regimes, root growth, physiological characteristics

## Abstract

In order to explore the effects of different irrigation methods on the physiological characteristics of mulch-free cotton in southern Xinjiang, the following experiments were carried out: (1) Different irrigation amount test: 300, 375, 450, 525, and 600 mm (represented by W1, W2, W3, W4, and W5) and a control (450 mm for film-covered cotton, represented by WCK) were set. (2) Drip irrigation frequency test: drip irrigation 12, 10, 8, and 6 times during the growth period (expressed by P12, P10, P8, and P6). Soil water dynamics, root distribution dynamics, chlorophyll fluorescence, leaf area index (LAI), SPAD (chlorophyll density), stress enzyme activities, and MDA (malondialdehyde) content were observed. The results showed that the average maximum change range of soil water content in the cotton field without film mulching was ±17.7%, which was 1.35 times higher than that in the cotton field with film mulching. Compared with cotton with film mulching, the root distribution characteristics of mulch-free cotton in the surface soil (0–20 cm) and the periphery (30 cm from the main root) decreased by 33.55–74.48% and 14.07–102.18%, respectively, while the root distribution characteristics in the deep layer (40–60 cm) increased by 49.62–242.67%, its average leaf green fluorescence parameters decreased by 9.03–50.44%, the activities of protective enzymes (SOD: superoxide dismutase, POD: peroxidase) decreased by 3.36–3.58%, the SPAD value decreased by 5.55%, and the MDA content increased by 3.17%, indicating that mulch-free cotton reduced the physiological function of cotton leaves, and the yield decreased by 42.07%. In the mulch-free treatments, the average root growth indexes were W2 > W3 > W4 > W5 > W1 and P12 > P10 > P8 > P6, and there was little difference between W2 and W3 and P12 and P10. With the increase in irrigation water and irrigation frequency, the initial fluorescence (F_0_) of leaves in each period of mulch-free cotton showed a downward trend, and the maximum fluorescence (F_m_), variable fluorescence (F_V_), maximum photochemical efficiency (F_V_/F_m_), potential photochemical activity of PS II (F_V_/F_0_), electron transfer of PS II (F_m_/F_0_), and photosynthetic performance index (PIABS) showed an upward trend. In all water treatments, W3 and P12 had the highest SPAD value, protective enzyme activity, and the lowest MDA content, which was significantly different from other treatments except W4 and P10. The yield order of different treatments was W3 > W4 > W5 > W2 > W1, and the difference between W3 and W4 was not significant, but significant with W2 and W1. The irrigation frequency test was P12 > P10 > P8 > P6, and there was no significant difference between P12 and P10. We find that in the mulch-free treatment, all indicators of W3, W2, P12, and P10 were relatively high. It can be concluded that no mulching has a certain impact on cotton root distribution and leaf physiological function. When the irrigation amount is 450–525 mm and irrigation times is 10–12, it is beneficial for promoting root growth and plays a role in leaf physiological function, and the water use efficiency (WUE) is high, which can provide reference for the scientific water management of mulch-free cotton in production practice.

## 1. Introduction

Xinjiang is the largest cotton planting area in China, with a planting area of 2.4479 million hectares and a total production of 5.686 million tons in 2024, accounting for 86.25% and 62.25% of the country’s total, respectively. Southern Xinjiang is rich in light and heat resources, which is very suitable for cotton production, and its planting area accounts for more than 65% of the whole of Xinjiang. In production, people generally use plastic film mulching cultivation, and the planting area of plastic film mulching is more than 98%. Plastic film mulching cultivation has become a key measure for a high and stable yield of cotton in southern Xinjiang [1]. Although plastic film mulching can significantly optimize soil ecological conditions, promote cotton growth, and increase yield, there are also major problems, mainly reflected in the difficulty of residual film recovery; the use of manual recovery is costly and difficult to implement in production. Although mechanical recovery is fast and low-cost, the recovery rate is not high. The long-term film mulching cultivation and the lack of effective residual film recovery methods have caused the accumulation of residual film in the field year by year, resulting in pollution and serious damage to the soil ecology [2]. Therefore, the implementation of mulch-free cultivation is the fundamental way to solve white pollution [3]. At present, people have cultivated new cotton varieties suitable for mulch-free cultivation, laying the foundation for mulch-free cultivation [4]. However, there are some practical problems in the mulch-free cultivation of cotton, such as slow emergence, late development, low yield, and low water and fertilizer utilization efficiency, which seriously restrict the application of mulch-free cultivation [5]. Compared with cotton with film mulching, cotton without film mulching has obvious differences in moisture, nutrient ecology, and cotton growth physiology [6]. How to carry out reasonable water and fertilizer management, regulate the coordinated growth of cotton root and crown, stimulate the adverse physiological activity of cotton yield formation, and improve the growth performance of cotton is the key to the formulation of cotton planting technology without film mulching. Water stress has an important impact on the growth physiology of cotton, which changes the balance of roots and shoots, antioxidant enzyme activity [7,8], and chlorophyll fluorescence kinetic parameters in cotton, reduces the photosynthetic performance of leaves, and has adverse effects on yield material synthesis and transportation. The soil water threshold for plant growth is generally 60–90 θ f (θ f is the field capacity). When the soil water is between 60% and 75% θ f and 75% and 90% θ f, there is a certain water stress. When the stress is small, plants produce a physiological compensation effect [9], which has little effect on growth, but when the stress is aggravated, the fluorescence, photosynthetic electron transfer activity, and antioxidant enzyme activity in plants change [10,11], which affects the physiological function of crops and is adverse to normal growth. Yang et al. [12] believed that severe water deficit reduced the photosynthetic performance of cotton leaves by 47.1%, and the maximum photochemical efficiency (F_v_/F_m_) and photochemical quenching coefficient (qP) decreased, while the non-photochemical quenching coefficient (qN) increased. Only by performing appropriate water management and fully meeting the physiological function of crops can we ensure a high yield and high efficiency of crops. There are many studies on the physiological response of cotton to water [13], but there are few studies on mulch-free cotton in extreme arid areas. This experiment carried out investigations on the effects of different irrigation amounts and frequencies on the root and crown and leaf physiological characteristics of mulch-free cotton, revealed the physiological mechanism of the corresponding water conditions of mulch-free cotton, and provides a theoretical basis for the formulation of an irrigation schedule for the efficient growth of mulch-free cotton.

## 2. Materials and Methods

### 2.1. Overview and Management of Test Area

The experiment was conducted in the net-house of the Agricultural Experiment Station of Tarim University, situated on the northwest edge of the Tarim Basin (40°33′ N, 81°16′ E, and elevation 1012.2 m). This location represents a typical extremely arid desert region, characterized by an average annual temperature of 11.2 °C, an accumulated temperature of ≥10 °C reaching 4076.2 °C, an average annual precipitation of 53.6 mm, an average annual evaporation of 1988.4 mm, and an average annual relative humidity consistently below 55%. It belongs to a typical inland climate in the warm temperate zone. The soil in the experimental field is composed of sandy loam soil, with a soil bulk density of 1.39 g·cm^−3^ in the 0–40 cm soil layer. The field water holding capacity stands at 23.8% (by weight), the wilting coefficient is 10.7%, the groundwater level is approximately 8.0 m, the content of soil organic matter is 10.25 g·kg^−1^, total nitrogen is 0.09%, alkali hydrolyzed nitrogen is 49.27 mg·kg^−1^, available phosphorus is 32.01 mg·kg^−1^, and available potassium is 214.10 mg·kg^−1^.

The irrigation regime test was conducted in 2022 and 2023. The plot area was 4.5 m × 10 m = 45 m^2^, and each area was separated by an impermeable plate (PVC polyester plate) with an isolation depth of 80 cm to prevent leakage. Before sowing, the soil was irrigated 180 mm in spring to store soil moisture. After a week, 600 kg·hm^−2^ of nitrogen, phosphorus, and potassium ternary compound fertilizer (NPK 19-17-6) was evenly applied and turned 25 cm deep. ‘Zhongmian 619’ (Cotton Research Institute of the Chinese Academy of Agricultural Sciences) was selected as the test material. On 5 April 2022 and 11 April 2023, the cotton was sown in the mode of no film deepening on-demand (sowing depth of 3.5 cm) and (66 + 10) × 10 cm mechanized cotton with shallow burying in the drip irrigation belt. The model of one film, four rows, and two tubes was adopted, and the theoretical number of plants was 239,200 plants·hm^−2^. The drip irrigation belt was placed in the middle of the narrow row and buried shallowly at 3 cm. In order to control weeds, 2250 mL·hm^−2^ of 33% pendimethalin EC (produced by Wuhan Jiyesheng Chemical Co., Ltd., Wuhan, China) was sprayed before sowing, and 300 kg·hm^−2^ of water was mixed. In order to maintain the weed control effect, 3–5 cm of soil was mixed after spraying. During the growth period, urea 600 kg·hm^−2^, high-phosphorus ternary compound water-soluble fertilizer (NPK 10-30-10+TE) 300 kg·hm^−2^ and high-potassium ternary compound water-soluble fertilizer (NPK 12-8-30+TE) 225 kg·hm^−2^ were applied with water drops. The plants were topped in mid July, and the chemical control was carried out three times at the full bud stage, the first flowering stage, and the full flowering stage. The dosage of 98% mepiquat wettable powder was 22.5 g·hm^−2^, 37.5 g·hm^−2^, and 120 g·hm^−2^, respectively. The irrigation water volume was recorded by the water meter in the community.

### 2.2. Test Materials and Design

Setup for 2 tests. The first experiment was the treatment of different drip irrigation water volume. Based on the drip irrigation quantity and time in production practice, after emergence, the irrigation amount should be increased or decreased according to the recommended irrigation amount, and five irrigation quotas were set, 300 mm, 375 mm, 450 mm, 525 mm, and 600 mm, with 450 mm of irrigation under the film as the control, and named as W1, W2, W3, W4, W5, and WCK, respectively. There were 6 treatments in the experiment, repeated 3 times, giving a total of 18 cells. Drip irrigation was conducted 9 times at the pregnant bud stage, the early bud stage, the full bud stage, the initial flowering stage, the full flowering stage, the late flowering stage, the early boll stage, full boll stage, and the initial boll opening stage according to the proportion of 0.1:0.1:0.12:0.12:0.14:0.12:0.1:0.1. See Table 1 for the specific allocation.

The second experiment was different drip irrigation frequency treatment. After emergence, four drip irrigation frequencies were set: 12 times, 10 times, 8 times, and 6 times, represented by P12, P10, P8, and P6. The drip irrigation quota was 450 mm. The experiment consisted of 4 treatments, repeated 3 times for a total of 12 cells. According to the law of cotton water demand, with more in the middle stage and less in the early and late stages, the water distribution in each growth period was carried out. The distribution of water in each growth period is shown in Table 2.

### 2.3. Observation Indicators and Measurement Methods

Soil water content (SWC): A comprehensive soil parameter sensor (Weihai Jingxun Changtong Electronic Technology Co., Ltd., Weihai, China) was used to monitor the soil moisture content of each plot. The sensor was installed 38 cm away from the drip irrigation belt, buried 20 cm, 40 cm, 60 cm, and 80 cm deep, and the relative value of water content in different soil layers was read. The drying method was used to establish a functional relationship between the reading (y) and the field moisture content (x), y = 92.065 − 96.95 × (R^2^ = 0.9121), to calculate and average the water content of each soil layer.

Root index: the root drill method was used to drill the root soil of the 0–20 cm, 20–40 cm, and 40–60 cm soil layers at 10 cm, 20 cm, and 30 cm away from the cotton plant at the bud stage, full bloom stage, boll stage, and boll opening stage, respectively. The roots were washed with clean water in a 200-mesh gauze and then selected. The WinRhizo root analysis system(v. 2020a; Regent Instruments, Inc., Quebec City, QC, Canada) was used to extract root length (RL), root surface area (RSA), root volume (RV), root average diameter (RAD), and other indicators, and then the root was dried and weighed (RG), and the root length density (RLD) and root weight density (RWD) were calculated according to the following formula:

RLD (mm · m^−3^) = RL/V    RWD (g · m^−3^) = RG/V

In the above formula, RL is the root length, RG is the root dry weight, and V is the root drill volume.

Chlorophyll fluorescence characteristics: During the main growth period of cotton, clear and cloudless weather was selected to measure the main stem leaves of cotton. Before measurement, the leaves were dark-treated for 30 min, and then the Yaxin-1105 chlorophyll fluorescence analyzer (Beijing Yaxin Liyi Technology Co., Ltd., Beijing, China) was used to measure the rapid chlorophyll fluorescence induction kinetic parameters of leaves under regular dark adaptation, including initial fluorescence (F_0_), maximum fluorescence (F_m_), variable fluorescence (F_V_), potential photochemical activity of PS II (F_V_/F_0_), electron transfer of PS II (F_m_/F_0_), maximum photochemical efficiency (F_V_/F_m_), and the photosynthetic performance index based on light absorption (PIABS).

Group Leaf Area Index (LAI): 2 consecutive plants were cut from each plot at different stages, all green leaves were picked and laid flat on white paper with no overlap facing down, the label board (black cardboard 10 cm × 10 cm) and plot number were placed in a corner of the white paper, a frontal photo with more than 8 million pixels (based on complete framing) was taken, the green leaf area was calculated using digital image processing technology [14], and then the group leaf area index (LAI) was calculated based on the total number of plants.

Chlorophyll value (SPAD): 10 cotton plants were randomly selected from each plot, and the relative chlorophyll value of the largest functional leaf on the main stem was measured using a 502 SPAD meter(Konica Minolta Co., Ltd., Tokyo, Japan), and the average value was taken.

Functional leaf antioxidant enzyme activity and malondialdehyde (MDA) content: An ELISA assay kit (Wuhan Jilide Biotechnology Co., Ltd., Wuhan, China) was used to determine the stress enzyme activities, such as peroxidase (POD) and superoxide dismutase (SOD), as well as MDA content in cotton functional leaves using a double-antibody one-step sandwich enzyme-linked immunosorbent assay.

Yield and water use efficiency (WUE) index: The final yield (kg·hm^−2^) was converted by manual picking and the statistics of actual yield in each plot. WUE (kg·hm^−2^·mm^−1^) = seed cotton yield/irrigation amount.

## 3. Results and Discussion

### 3.1. Effects of Different Water Treatments on Soil Water Content in Mulch-Free Cotton Field

**Figure 1:**
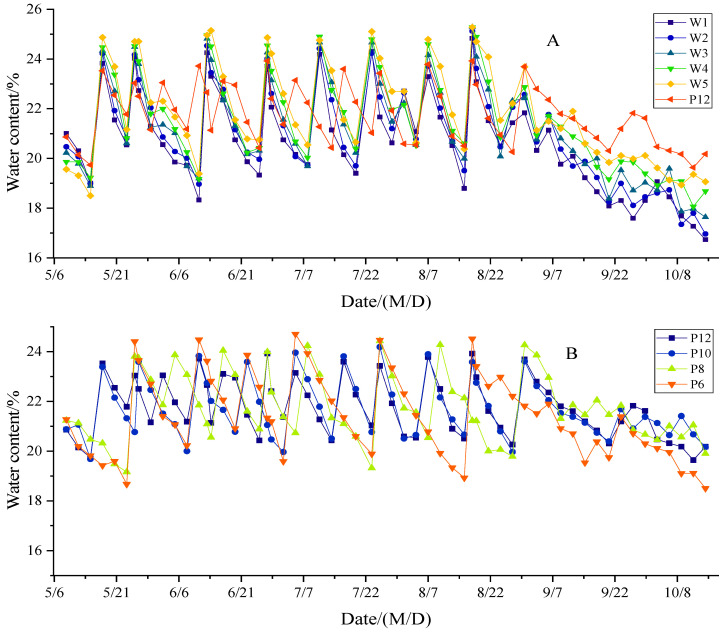
Dynamics of average water content in 0–80 cm soil layer under different irrigation quantities (**A**) and irrigation frequencies (**B**). W1, W2, W3, W4 and W5 represent irrigation of 300, 375, 450, 525 and 600 mm for mulch-free cotton, and WCK represents irrigation of 450 mm for filmed cotton.P12, P10, P8, and P6 represent drip irrigation for 12, 10, 8, and 6 times, respectively.

See Figure 1 for the change in soil water content in each treatment. With the development of the growth process, the soil water content showed a tortuous change with the irrigation activities. The soil water content increased sharply on the day of drip irrigation, and then gradually decreased, and fluctuated once per drip irrigation. After the final irrigation, the fluctuation in soil water content becomes smaller and its value gradually decreases. There were some differences in the average water content of the 0–80 cm soil layer between different water treatments. The average water content of W1, W2, W3, W4, W5, and WCK was20.73%, 21.06%, 21.33%, 21.66%, 22.01%, and 21.61% (CV = 2.15%), respectively. The average water content of P12, P8, P8, and P6 was 21.14%, 21.21%, 21.20%, and 20.77% (CV = 0.50%), respectively. This showed that increasing the irrigation amount and frequency could increase soil water content to a certain extent, and the effect of the increase in irrigation amount on soil water content was significantly higher than that of irrigation frequency. From the fluctuation range of average water content in the 0~80 cm soil layer, the maximum fluctuation range of mulch-free cotton was ±19.5%, with an average of ±17.7%, which was higher than ±16.1% of WCK, indicating that film-free mulching aggravated the change in soil moisture. The variation range of soil water content in W3 and W4 was ±17.1% and ±16.6%, which was not different from that in WCK. The changes in soil water content in the P12, P8, P8, and P6 treatments were ±15.7%, ±17.1%, ±17.3%, and ±20.5%, respectively, and showed a decreasing trend with the increase in irrigation times. It can be seen that increasing irrigation frequency is conducive to reducing the changes in soil water content, which is beneficial to cotton growth.

### 3.2. Effects of Different Water Treatments on Root Growth of Mulch-Free Cotton

**Figure 2:**
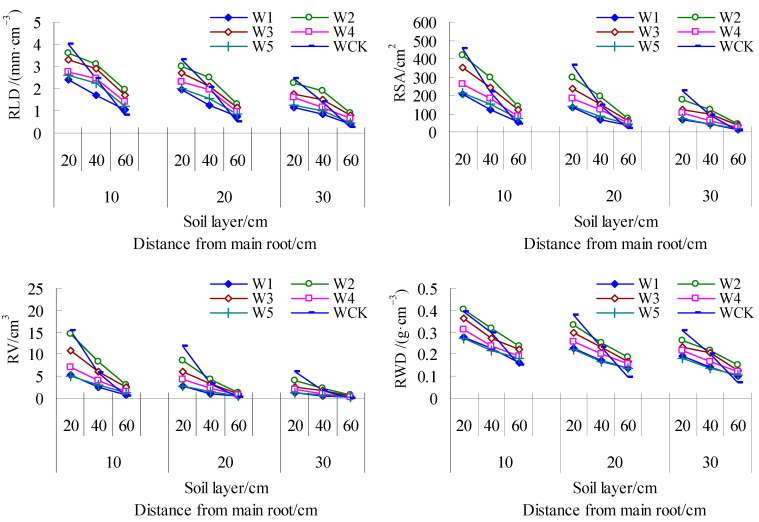
Spatial distribution dynamics of mulch-free cotton roots in soil under different irrigation treatments.W1, W2, W3, W4 and W5 represent irrigation of 300, 375, 450, 525 and 600 mm for mulch-free cotton, and WCK represents irrigation of 450 mm for filmed cotton, respectively.

It can be seen from Figure 2 that the characteristic values of the root growth of mulch-free cotton decrease with the deepening of the soil layer and the increase in distance from the main root, and the values among the irrigation amount treatments are W2 > W3 > W4 > W5 > W1, in which the average RLD, RSA, RV, and RWD of W2 are the largest, and the average characteristic values of W2 in each soil layer and at different distances from the main root are also the largest, followed by W3 and W1. Compared with other treatments, the RLD of W2 periphery (30 cm from the main root) and middle layer (20–40 cm soil layer) increased by 24.57–106.67% and 14.99–94.55%, the average proportion of RSA in total RSA increased by 1.64–3.41 and 0.49–4.09 percentage points, and the average proportion of RV in total RV increased by 1.00–2.54 and 0.09–4.52 percentage points. The RLD of W3 increased by 19.41–65.91% and 17.69–69.19% compared with other treatments (except W2). The proportion of average RSA in total RSA in W3 deep layer (40–60 cm soil layer) was 0.27–1.20 percentage points higher than that in other treatments, and the proportion of average RV in total RV was 1.11–2.12 percentage points higher than that in other treatments. The average RLD of WCK was the largest, but there was no significant difference with W3. The total RSA and average RV and RWD of WCK were 1564.41 cm^2^, 44.98 cm^3^, and 0.24 g·cm^−3^, respectively, which were second only to W2. In addition, the root distribution of WCK at 30 cm away from the main root was second only to W2, and its RLD, RSA, RV, and RWD increased by 1.89–69.05%, 26.27–156.36%, 12.70–296.87%, and 2.31–34.14% compared with the treatment without a membrane (except W2), respectively. The root distribution of WCK was the least in the 40–60 cm soil layer, and its RLD, RSA, RV, and RWD decreased by 44.88–166.90%, 61.69–303.00%, 72.05–484.18%, and 25.41–80.92% compared with the treatment without a membrane, and increased by 10.09–76.45%, 16.60–152.88%, 22.43–263.99%, and 8.58–60% in the 0–20 cm soil layer. Therefore, mild water deficit (W2) and suitable water (W3) can expand the distribution range of cotton roots in soil; excessive drought (W1) is unfavorable to root growth, while film mulching planting mainly promotes the development of cotton surface and peripheral roots, which is not conducive to deep water and nutrient absorption.

**Figure 3:**
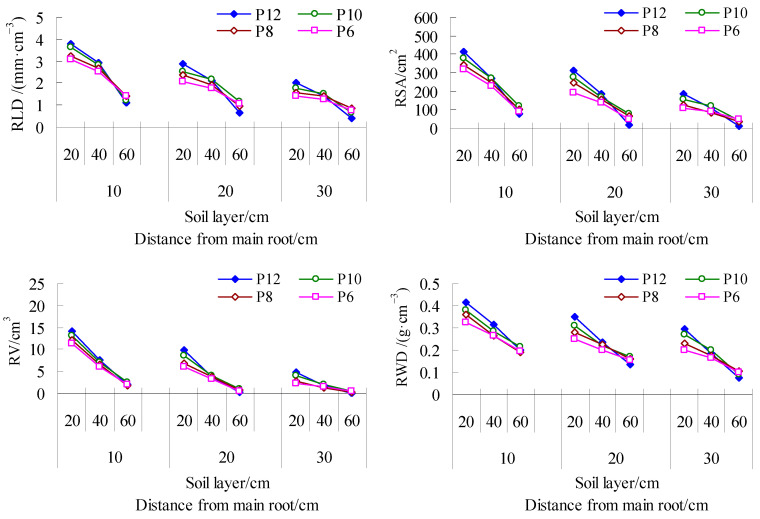
Spatial distribution dynamics of mulch-free cotton roots in soil under different irrigation frequencies. P12, P10, P8, and P6 represent drip irrigation for 12, 10, 8, and 6 times, respectively.

It can be seen from Figure 3 that the average RLD and RSA of different soil layers during the whole period between the irrigation frequency treatments are P10 > P12 > P8 > P6, and P10 increases by 0.35% and 2.37% compared with P12, with little difference, and increases by 6.02% and 15.28% and 13.36% and 28.30% compared with P8 and P6, with obvious difference. The RV and RWD are P12 > P10 > P8 > P6, and P12 increases by 2.91% and 2.76% compared with P10, with little difference, and increases by 21.23% and 10.82% and 32.11% and 18.74% compared with P8 and P6, with obvious difference. It can be seen that the P12 and P10 treatments can effectively increase the number of roots, which is beneficial to root growth. From the perspective of root spatial distribution, each characteristic value in the 40–60 cm soil layer was P10 > P8 > P6 > P12, and that 30 cm away from the main root was P10 > P12 > P8 > P6, indicating that the P10 treatment had deeper and more peripheral roots, and the root range was the largest.

### 3.3. Effects of Different Water Treatments on Chlorophyll Fluorescence Induction Kinetic Parameters of Mulch-Free Cotton

Chlorophyll fluorescence is an important component of photosynthesis and can reflect changes in most photosynthetic processes [15]. As shown in Table 3, with the increase in irrigation water, all chlorophyll fluorescence parameters of mulch-free cotton showed an upward trend, except for F_0_, which showed a downward trend. Among them, the F_0_ of W1 was the largest, significantly higher than that of W5 during the bud, flower, boll, and boll opening stages, with increases of 25.05%, 32.67%, 12.55%, and 18.16%, respectively. The F_m_ and F_V_ of W5 were the largest during the bud, flower, and boll opening stages, with increases of 14.94%, 15.45%, and 30.09% and 16.32%, 20.52%, and 34.71% compared to W1. The F_m_ and F_V_ of W4 were the largest during the boll stage, with increases of 8.78% and 15.13% compared to W1. The F_V_/F_m_, F_V_/F_0_, F_m_/F_0_, and PIABS of W5 were the largest during all periods, with increases of 5.48–14.06%, 23.39–34.92%, 10.74–25.49%, and 55.78–116.59% compared to W1. It is evident that drought stress has a significant impact on the chlorophyll fluorescence parameters of mulch-free cotton, with PIABS being the most affected (the coefficient of variation CV for each treatment is 19.37–27.10%). Appropriate higher irrigation levels (W4, W5) are beneficial to improve the photosynthetic fluorescence characteristics of mulch-free cotton and promote its material production capacity. Comparing the average values of each parameter of mulch-free cotton with WCK (mulched cotton), the F_0_ of WCK is not significantly different from that of mulch-free cotton, while its F_m_, F_V_, F_V_/F_m_, F_V_/F_0_, F_m_/F_0_, and PIABS are increased by 14.49–20.18%, 22.13–36.67%, 8.87–12.25%, 24.72–44.33%, 15.22–36.08%, and 37.82–132.97% compared to the average values of mulch-free cotton. It is clear that non-mulching has a significant impact on the leaf photosynthetic fluorescence parameters, with PIABS and F_V_/F_0_ being the most affected.

As the frequency of irrigation increases, all fluorescence parameters except for F_0_ show an increasing trend during various growth stages. Among them, the F_0_ value is the highest for P6, and it significantly differs from other treatments except for P8 during the bell stage. F_m_, F_V_, F_V_/F_m_, F_V_/F_0_, F_m_/F_0_, and PIABS all reach their maximum under P12, which is significantly higher than under P6, with increases ranging from 8.48% to 42.80%, 13.71% to 43.98%, 11.11% to 27.66%, 28.81% to 64.29%, 15.14% to 45.27%, and 42.51% to 163.64%, respectively. This indicates that higher irrigation frequencies can improve leaf photosynthetic fluorescence parameters and enhance leaf photosynthetic function. From the perspective of the CV changes of each parameter under different treatments at various stages, PIABS has the highest variation, ranging from 15.37% to 44.75%, followed by F_0_, which varies from 20.55% to 25.59%, while F_V_/F_m_ has the smallest variation, ranging from 4.85% to 10.18%. This suggests that the irrigation frequency mainly affects the photosynthetic performance index and the initial fluorescence intensity, with a smaller impact on the maximum photochemical efficiency (Table 4).

### 3.4. Effects of Different Water Treatments on the SPAD of Mulch-Free Cotton

**Figure 4:**
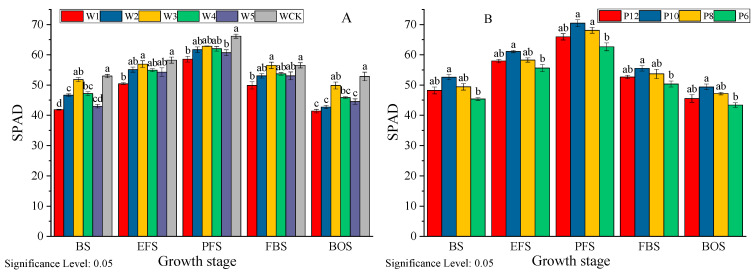
Effects of different irrigation quantities (**A**) and irrigation frequencies (**B**) on the relative chlorophyll content (SPAD) of mulch-free cotton. BS, EFS, PFS, FBS, and BOS, respectively, represent the budding stage, early flowering stage, peak flowering stage, full boll stage, and boll opening stage. Different lowercase letters in the same column indicate significant differences between different irrigation treatments at the same time (*p* < 0.05).

From Figure 4, it can be seen that the SPAD value of mulch-free cotton leaves shows a trend of first increasing and then decreasing with the growth process, reaching its maximum at the full flowering stage (88 days after seedling). In the irrigation experiment, the average SPAD value of WCK leaves was the highest, increasing by 2.86–19.78% compared to non-film cotton. The average SPAD value of cotton leaves without film treatment showed W3 > W4 > W2 > W5 > W1, with W3 significantly higher than W1, W5, and W2, a decrease of 1.60 compared to WCK, but the difference was not significant. In addition, from the perspective of the variation in SPAD values during the growth period (CV) of each treatment, W3 had the smallest variation (CV = 8.95%), which was not significantly different from WCK (CV = 9.66%). W1, W2, and W5 showed greater variation (CV = 14.56–14.84%), indicating that appropriate irrigation can promote the improvement of leaf function in non-film cotton and prevent premature aging of leaves, and that excessive or insufficient irrigation can be detrimental to leaf function. In the drip irrigation frequency test, the average SPAD value of the leaves showed P10 > P8 > P12 > P6, with P10 being 57.09, an increase of 11.01%, 4.99%, and 5.32% compared to P6, P8, and P12, respectively. The CV values of SPAD for each treatment ranged from 11.84 to 12.96%, indicating that appropriately higher irrigation times are beneficial for the development of leaf function.

### 3.5. Effects of Different Water Treatments on Leaf Area Index (LAI) of Mulch-Free Cotton

**Figure 5:**
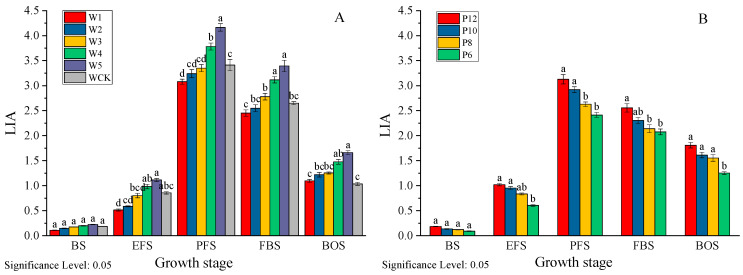
LAI dynamics of mulch-free cotton under different irrigation quantities (**A**) and irrigation frequencies (**B**). BS, EFS, PFS, FBS, and BOS, respectively, represent the budding stage, early flowering stage, peak flowering stage, full boll stage, and boll opening stage. Different lowercase letters in the same column indicate significant differences between different irrigation treatments at the same time (*p* < 0.05).

From Figure 5, it can be seen that the LAI of mulch-free cotton shows a trend of first increasing and then decreasing as the growth process progresses. It rapidly increases after the initial flowering period, reaches its maximum during the peak flowering period, and rapidly decreases after the bolling period. In the irrigation experiment, the LAI of all treatments of non-film cotton increased with the increase in irrigation water, showing W5 > W4 > W3 > W2 > W1. W5 increased by 20.52–60.37% compared to WCK at each stage. During the boll opening period, WCK experienced a sharp decrease in LAI due to its shallow root system and the cessation of irrigation. From the average value of LAI, there is not much difference between W3 and WCK, but its CV value for LAI is smaller than that of W1, W2, and WCK, indicating that appropriate irrigation can maintain good LAI dynamics and maintain the photosynthetic area in the later stage of the canopy. In the drip irrigation frequency experiment, the LAI of each treatment increased with the increase in drip irrigation frequency, showing a trend of P12 > P10 > P8 > P6 at all stages. P12 increased by 18.78–50.42% compared to P6. Due to fewer irrigation cycles, there is significant variation in LAI during each period in P6. It can be seen that increasing irrigation frequency is beneficial for the development of canopy leaf area in non-membrane cotton.

### 3.6. Effects of Different Water Treatments on Stress Biochemical Substances of Mulch-Free Cotton

#### 3.6.1. Effect on Malondialdehyde (MDA) Content

**Figure 6:**
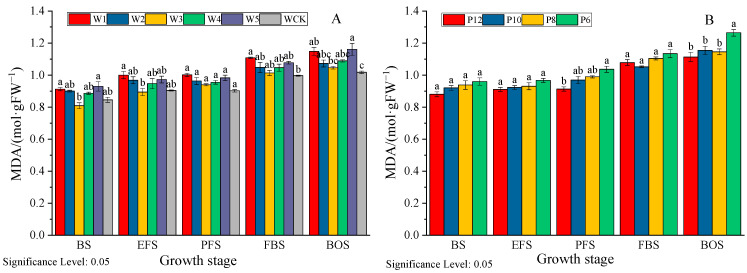
Effects of different irrigation quantities (**A**) and irrigation frequencies (**B**) on the MDA content of mulch-free cotton.BS, EFS, PFS, FBS, and BOS, respectively, represent the budding stage, early flowering stage, peak flowering stage, full boll stage, and boll opening stage. Different lowercase letters in the same column indicate significant differences between different irrigation treatments at the same time (*p* < 0.05).

From Figure 6, it can be seen that the MDA content in leaves shows an upward trend with the growth process, with an average increase of 24.31% during the boll open period compared to the bud period in each treatment. There is not much difference between the initial flowering period and the peak flowering period. The MDA content in mulch-free cotton leaves is generally higher than that in WCK, with an average value 4.97–8.42% higher in each treatment and stage compared to WCK, indicating that non-membrane coverage has certain adverse effects on cotton growth. The MDA content showed a trend of first decreasing and then increasing with the increase in irrigation volume among different irrigation volumes, manifested as W3 < W4 < W2 < W5 < W1. The MDA content of W3 was 0.94 mol·gFW^−1^ during the peak flowering period, which was not significantly different from W4, but decreased by 2.53%, 4.63%, and 6.57% compared to W2, W5, and W1, with an average decrease of 5.32%, 8.95%, and 9.95%, respectively. In the drip irrigation frequency experiment, the MDA content of each treatment showed a decreasing trend with the increase in drip irrigation frequency. P6 had the highest MDA content, which increased by 9.05–13.62% compared to P12. This indicates that appropriate water treatment (W3, W4) and increasing irrigation frequency are beneficial for reducing the harm of water stress in mulch-free cotton.

#### 3.6.2. Effect on the Activity of Stress-Protective Enzymes

**Figure 7:**
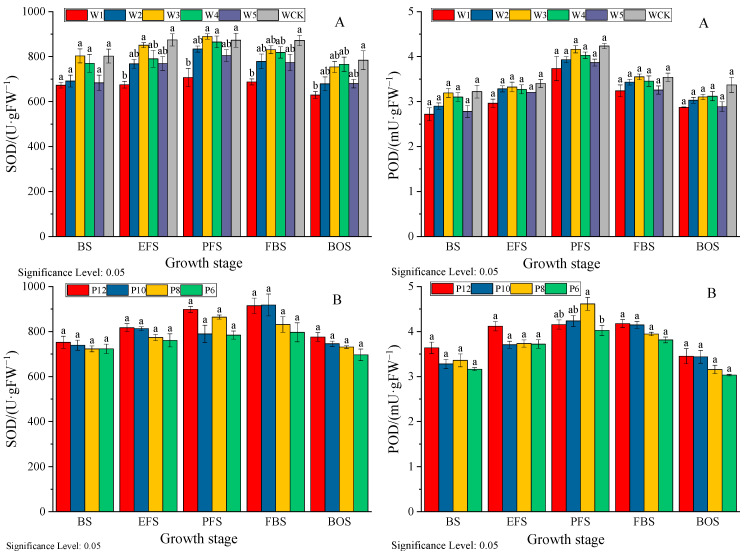
Effects of different irrigation quantities (**A**) and irrigation frequencies (**B**) on SOD and POD activities of mulch-free cotton. BS, EFS, PFS, FBS, and BOS, respectively, represent the budding stage, early flowering stage, peak flowering stage, full boll stage, and boll opening stage. Different lowercase letters in the same column indicate significant differences between different irrigation treatments at the same time (*p* < 0.05).

Superoxide dismutase (SOD) is an important antioxidant enzyme in the stress-protective enzyme system, playing a crucial role in cellular antioxidant and anti-aging. Peroxidase (POD) is an enzyme that removes peroxides from the plant body and can reduce the damage of reactive oxygen species to cotton plants. According to Figure 7, as the growth period progresses, the activity of various protective enzymes shows a trend of first increasing and then decreasing, with the highest activity occurring during the peak flowering period to the boll stage. The SOD and POD activities of mulch-free cotton were lower than those of WCK, and the average values of each irrigation treatment were 6.43–13.46% and 4.52–12.32% lower than those of WCK. The SOD and POD activities between different irrigation treatments were W3 > W4 > W2 > W5 > W1. The average SOD and POD activities of W3 were not significantly different from those of W4 (2.08–2.94%), but significantly different from those of W5 and W1, increasing by 11.23%, 22.44%, 8.35%, and 11.63%, respectively. The appropriate irrigation amount is beneficial for improving the activity of protective enzymes. Increasing the number of irrigation sessions is beneficial for improving SOD and POD activities, with the average values of P12 increasing by 3.81%, 6.00%, and 10.56% and 3.89%, 3.87%, and 10.06%, respectively, compared to P10, P8, and P6.

### 3.7. Effects of Different Water Treatments on Yield and WUE of Mulch-Free Cotton

**Figure 8:**
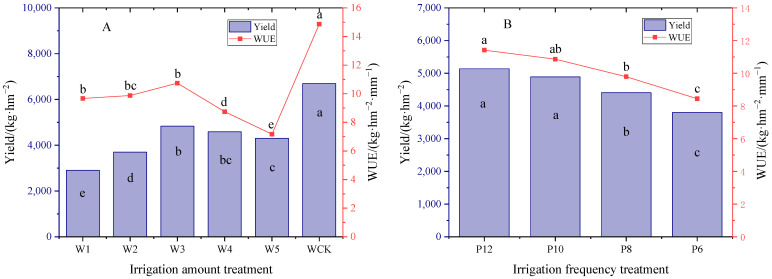
Effects of different irrigation quantities (**A**) and irrigation frequencies (**B**) on seed cotton yield and WUE of mulch-free cotton. W1, W2, W3, W4 and W5 represent irrigation of 300, 375, 450, 525 and 600 mm for mulch-free cotton, and WCK represents irrigation of 450 mm for filmed cotton. P12, P10, P8, and P6 represent drip irrigation for 12, 10, 8, and 6 times, respectively. The different lowercase letters indicate significant differences between treatments (*p* < 0.05).

According to Figure 8, among the different irrigation treatments, WCK had the highest yield and WUE, increasing by 38.5–130.7% and 38.48–130.7%, respectively, compared to the no-film treatment. With the increase in irrigation water, the yield and WUE of mulch-free cotton increased first and then decreased, and W3 was the largest, with a yield of 4833.25 kg·hm^−2^, which was not significantly different from the W4 treatment, but significantly higher than other treatments, with a WUE of 10.74 kg·hm^−2^·mm^−1^, which was not significantly different from the W2 treatment, but significantly higher than other treatments. In different irrigation frequency treatments, with the increase in irrigation frequency, the yield and WUE of mulch-free cotton gradually increased. The yield and WUE of P12 were 5183.71 kg·hm^−2^ and 11.42 kg·hm^−2^·mm^−1^, respectively, which had little difference with the P10 treatment, but were significantly higher than those of the P8 and P6 treatments. It can be seen that an appropriate irrigation amount and higher irrigation frequency are conducive to the efficient use of water and yield potential of mulch-free cotton.

### 3.8. Correlation Analysis Between Different Water Treatments and Root Indexes and Physiological Indexes

**Figure 9:**
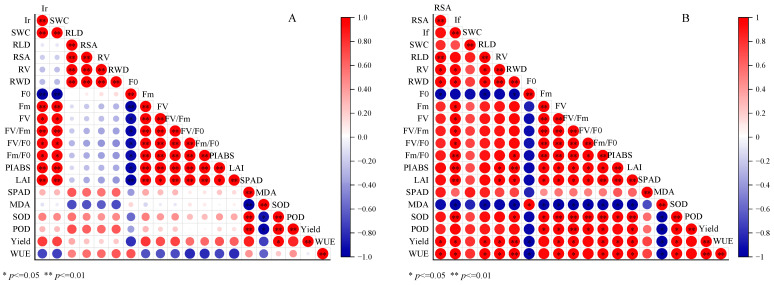
Correlation between soil moisture, root growth, physiological performance, yield, and WUE under different irrigation amounts (**A**) and irrigation frequencies (**B**). In the above figure, Ir: Irrigation volume, If: Irrigation frequency, SWC: Soil water content, RLD: Root length density, RSA: Root surface area, RV: Root volume, RWD: Root weight density, F0: Initial fluorescence, Fm: Maximum fluorescence, FV: Variable fluorescence, FV/Fm: Maximum photochemical efficiency, FV/F0: Potential photochemical activity of PSII, Fm/F0: Electron transfer of PSII, PLABS: Photosynthetic performance index based on light absorption, LAI: Leaf area index, SPAD: Relative chlorophyll content, MDA: Malondialdehyde content, SOD: Superoxide dismutase activity, POD: Peroxidase activity, Yield: Production, WUE: water use efficiency.

In order to further understand the relationship between water supply and root system and physiological indexes, correlation analysis of each index showed that (Figure 9), irrigation amount was significantly positively correlated with soil water content, chlorophyll fluorescence parameters (except F_0_), LAI, and yield, negatively correlated with root growth index, MDA, and WUE, and positively correlated with SPAD and protective enzymes (SOD, POD), but not significantly. Irrigation frequency was positively correlated with soil water content, root development index, chlorophyll fluorescence parameters (except F_0_), LAI, SPAD value, protective enzymes, yield, and WUE, and negatively correlated with F_0_ and MDA. Chlorophyll fluorescence parameters (except F_0_), LAI, SPAD value, and protective enzymes were significantly positively correlated with yield, while F_0_ and MDA were significantly negatively correlated with other indicators. It can be seen that properly reducing the amount of irrigation and increasing irrigation times have a good role in promoting the root growth of mulch-free cotton. Excessive irrigation can lead to vigorous aboveground growth and enhanced physiological activity, but affect the root growth and reduce WUE, which is unfavorable to the final effective yield formation and efficient water use. There was a weak positive correlation between root growth index and WUE and the yield of mulch-free cotton in different irrigation amount treatments, while there was a very significant positive correlation between root growth index and WUE and yield in different irrigation frequency treatments. It can be seen that the key to improving the yield and WUE of mulch-free cotton is to ensure a higher irrigation frequency and determine the appropriate irrigation amount to coordinate the balance between root cap growth and yield formation.

## 4. Discussion

The soil water content of the drip irrigation mulch-free cotton field in Southern Xinjiang showed a zigzag change with irrigation activities. The greater the amount of irrigation, the lower the irrigation frequency, and the greater the change range. The maximum and average variation in soil moisture content in the 0–80 cm soil layer of the non-film treatment were ±17.7% and ±6.49%, respectively, which were 1.35 and 1.17 times higher than those of the film treatment. Root growth is closely related to soil moisture [16]. In this experiment, the root distribution of cotton without film is narrower and deeper than that of cotton with film, and the root distribution range and root amount are the widest and the largest when the irrigation amount is 375–450 mm and the whole-period drip irrigation is about 10 times, which is different from that of cotton with film. Yang [17] and Luo et al. [18] believe that irrigation of 370–390 mm and irrigation 6–8 times is beneficial to the optimal distribution of cotton with film. It can be seen that no film mulching leads to dramatic changes in soil moisture content and increased evaporation in the field [19], which intensifies water consumption and is unfavorable to the efficient use of water and high yield of cotton [20]. Only increasing irrigation quantity and times properly can ensure the optimal growth of root system of mulch-free cotton.

Chlorophyll fluorescence is closely related to photosynthesis, reflecting the absorption, transmission, and conversion of light energy by leaves [21,22]. Fluorescence changes can reflect the situation of photosynthesis and heat dissipation [23,24]. Genty et al. [10] reported that drought had little effect on the chlorophyll fluorescence parameters of cotton. The results of this study indicate that water deficit has a significant impact on chlorophyll fluorescence parameters. The F_0_ of the water deficit treatment significantly increased, while the F_m_, F_V_, F_V_/F_m_, F_V_/F_0_, F_m_/F_0_, and PIABS indicators showed a decreasing trend with the deepening of water deficit. This is consistent with the research results of Xue et al. [25] and Dou et al. [26]. Abdulkadir et al. [27] believe that drip irrigation 7 times in film-covered cotton fields in Southern Xinjiang is beneficial for the development of leaf photosynthetic function. In this study, increasing the number of irrigation times can effectively reduce the degree of photoinhibition or damage to PS II complexes. The leaf fluorescence parameters of P12 reached a good level, which may be related to the ecological differences in soil moisture in the plot with or without film mulching. Maintaining a high drip irrigation frequency for non-film-covered cotton can effectively maintain the soil moisture content in the root zone and promote the leaf activity of cotton plants [28].

It is generally believed [29,30] that LAI increases with the increase in irrigation volume, and the time to reach its peak shifts later. When the water volume is too low, the population becomes smaller and the photosynthetic area decreases. As an antioxidant response indicator of plant membrane system stress perception, MDA content increases with the degree of water stress, while the activity of protective enzymes such as SOD and POD, which regulate the content of osmotic substances in the body, shows a decreasing trend [31,32]. In this study, both water deficit and excess water had negative effects on the LAI, MDA content, and protective enzymes of mulch-free cotton. The CV of LAI, MDA content, and SOD and POD activity changes during cotton growth in the water deficit treatment (W1 and W2) were 84.54–87.61%, 9.10–10.09%, 6.16–8.61%, and 12.15–13.29%, respectively, which were higher than those in the multi-water treatment (W4 and W5) (77.10–78.17%, 8.29–9.10%, 5.11–7.67%, and 11.23–12.86%). This indicates that water deficit has a greater impact on the physiological functions of mulch-free cotton than excessive water, which is also one of the reasons why mulch-free cotton has higher water requirements [33]. In addition, it was found in this study that the appropriate irrigation amount treatments (W3 and W4) increased LAI and SOD and POD activity by 4.90–17.64% and 21.07–42.68%, 3.99–4.99% and 2.40–6.53%, and 0.29–8.66% and 2.45–8.08%, respectively, compared to WCK during the boll and opening stages. This indicates that the population growth of mulch-free cotton is higher in the later stage, which may be related to the more developed root system of mulch-free cotton [34], and this is also one of the reasons why non-membrane cotton is less prone to premature aging [35].

The test found that compared with WCK, the average value of chlorophyll fluorescence parameters in the whole period of W3 and W4 treatment of mulch-free cotton decreased; except for F_0_, other parameters such as F_m_, F_V_, F_V_/F_m_, F_V_/F_0_, F_m_/F_0_, PIABS were reduced by 20.58%, 33.21%, 10.19%, 48.22%, 36.77%, and 70.68%, the leaf SPAD value was reduced by 5.55%, protective enzyme activity was reduced by 3.36–3.58%, MDA content increased by 3.16%, and the yield decreased by 42.07%. It can be seen that the physiological function of the leaves of cotton without film is reduced to a certain extent compared with that with film, which may be related to the fact that the field water ecological conditions of cotton without film mulching are not as good as those with film mulching [36], resulting in a significant decline in yield. There are also studies that show that there is no significant difference between the yield of cotton cultivated without film and that of cotton with film [37], which may be related to the cultivation method and needs further study.

## 5. Conclusions

The physiological function of the roots and leaves of mulch-free cotton were the strongest at the flowering and bolling stage, and the peak value of its function was later than that of film-covered cotton. The physiological functions of the leaves of mulch-free cotton were lower than those of film-covered cotton, and were greatly affected by water conditions. The degree of influence was greater for irrigation water than irrigation frequency.

Compared with the cotton field with film mulching, the range of water change in the mulch-free cotton field increased, the horizontal distribution of roots narrowed, and the vertical distribution deepened. Properly increasing the amount and frequency of irrigation could promote the root development of mulch-free cotton and improve the chlorophyll fluorescence parameters of leaves, which mainly showed that the root characteristic values (RLD, RSA, RV, and RWD), PIABS, and F_v_/F_0_ increased significantly. The root growth characteristic value and leaf SPAD value of W3 and P12 were larger, and had little difference with W2, W4, and P10. The LAI of W3 had little difference with WCK, and the change difference of each growth period was small. The MDA content of W3, W4, and P12 was lower, the activities of SOD and POD were higher, and their yield and WUE were the largest, indicating that the irrigation amount of 450–525 mm and irrigation frequency of 10–12 times were beneficial to the physiological function of mulch-free cotton leaves, which could be used as a reference for water management in production practice.

## Figures and Tables

**Table 1:** Experimental setup for different irrigation amounts (mm).

Treatment	Pregnant Bud Stage (5–18)	Early Bud Stage (5–26)	Full Budding Period (6–14)	Early Flowering Stage (6–28)	Full Blooming Period (7–10)	Late Blooming Stage (7–22)	Early Boll Stage (8–5)	Full Boll Stage (8–18)	Early Boll Opening Stage (8–28)	Irrigation Quota
W1	30	30	30	36	36	42	36	30	30	300
W2	37.5	37.5	37.5	45	45	52.5	45	37.5	37.5	375
W3	45	45	45	54	54	63	54	45	45	450
W4	52.5	52.5	52.5	63	63	73.5	63	52.5	52.5	525
W5	60	60	60	72	72	84	72	60	60	600
WCK	45	45	45	54	54	63	54	45	45	450

Note: The numbers in parenthesis in the header are the date, M–D. The average temperature of the 0–80 cm soil layer in each treatment was 21.10–22.13 °C in the whole period, which had a certain downward trend with the increase in irrigation water, but the difference was not significant (coefficient of variation CV = 1.76%).

**Table 2:** Experimental setup for different irrigation frequencies (mm).

Treatment	Early Bud Stage (6–10)	Full Budding Period (6–17)	Full Budding Period (6–20)	Early Flowering Stage (6–24)	Early Flowering Stage (6–27)	Early Flowering Stage (7–1)	Early Flowering Stage (7–7)	Full Flowering Period (7–11)	Full Flowering Period (7–13)
P12	27	27	0	36	0	36	45	0	45
P10	36	0	45	0	0	45	0	54	0
P8	0	45	0	0	54	0	54	0	63
P6	0	63	0	0	0	72	0	81	0
**Treatment**	**Full Flowering Period** **(7–19)**	**Full Flowering Period** **(7–21)**	**Full Flowering Period** **(7–26)**	**Early Boll Stage** **(8–2)**	**Early Boll Stage** **(8–10)**	**Full Boll Stage** **(8–12)**	**Full Boll Stage** **(8–18)**	**Early Boll Opening Stage** **(8–28)**	**Irrigation Quota**
P12	45	0	45	36	36	0	36	36	450
P10	54	0	54	45	45	0	36	36	450
P8	0	63	0	63	0	54	0	54	450
P6	81	0	0	81	0	0	72	0	450

Note: The numbers in parenthesis in the header are the date, M–D. The average temperature of the 0–80 cm soil layer in each treatment was 21.71–21.97 °C, which had a certain downward trend with the increase in irrigation frequency, but the difference was not significant (the coefficient of variation CV = 0.50%).

**Table 3:** Effect of irrigation amount on chlorophyll fluorescence parameters of mulch-free cotton.

Period	Process	F_0_	F_m_	F_V_	F_V_/F_m_	F_V_/F_0_	F_m_/F_0_	PIABS
Bud stage	W1	216.33 a	723.00 c	514.73 d	0.65 d	2.18 d	3.26 cd	2.45 d
	W2	206.00 ab	724.00 c	524.41 cd	0.67 cd	2.32 cd	3.18 d	2.99 c
	W3	194.33 bc	740.67 c	535.64 c	0.69 bc	2.26 d	3.32 cd	3.24 c
	W4	188.00 c	824.67 b	535.86 c	0.71 b	2.55 bc	3.49 bc	3.38 c
	W5	173.00 d	831.00 b	598.73 b	0.71 b	2.69 b	3.61 b	4.38 b
	WCK	198.33 bc	910.67 a	740.57 a	0.77 a	3.41 a	4.43 a	7.66 a
Blossom period	W1	246.33 a	733.67 d	545.91 d	0.72 c	2.52 d	3.57 d	5.54 c
	W2	219.00 b	754.33 cd	562.91 cd	0.73 c	2.79 c	3.69 cd	5.20 c
	W3	214.00 bc	773.67 c	580.93 cd	0.75 bc	2.84 c	3.82 cd	6.42 b
	W4	193.00 d	792.33 c	602.00 c	0.74 c	3.00 c	3.87 c	6.61 b
	W5	185.67 d	847.00 b	657.94 b	0.78 b	3.40 b	4.48 b	8.63 a
	WCK	210.67 c	937.67 a	740.25 a	0.81 a	4.20 a	5.04 a	9.09 a
Boll stage	W1	275.00 a	819.67 c	568.27 d	0.73 bc	2.73 c	3.52 d	6.34 d
	W2	264.00 b	849.33 bc	596.90 c	0.69 b	2.48 c	3.74 c	7.21 d
	W3	256.00 bc	868.33 bc	609.99 c	0.74 b	2.89 bc	3.83 c	8.49 c
	W4	247.33 cd	891.67 b	654.23 b	0.74 b	2.88 bc	3.89 c	8.53 c
	W5	244.33 d	855.33 bc	643.71 b	0.77 ab	3.48 ab	4.31 b	10.55 b
	WCK	258.00 b	981.00 a	750.63 a	0.80 a	4.04 a	5.25 a	11.79 a
Lint stage	W1	201.67 a	585.00 d	403.58 c	0.64 d	1.99 d	2.98 d	2.23 d
	W2	189.67 b	612.33 cd	423.48 c	0.67 cd	2.02 d	3.03 d	3.16 c
	W3	182.67 bc	726.33 b	522.13 b	0.69 bc	2.32 c	3.26 c	4.08 b
	W4	176.67 cd	639.67 c	426.30 c	0.70 bc	2.22 c	3.22 c	3.84 b
	W5	170.67 d	761.00 a	543.65 b	0.73 ab	2.49 b	3.48 b	4.83 a
	WCK	183.67 bc	792.33 a	604.15 a	0.76 a	2.82 a	3.68 a	5.00 a

Note: F_0_ is the initial fluorescence intensity of the leaf; F_m_ is the maximum fluorescence intensity; F_V_ is the variable fluorescence intensity; F_V_/F_m_ is the maximum photochemical efficiency of PS II; F_V_/F_0_ is the potential photochemical activity of PS II; F_m_/F_0_ is the electron transport status of PS II; PIABS reflects the photosynthetic performance index based on absorbed light energy; different lowercase letters in the same column indicate significant differences between treatments (*p* < 0.05).

**Table 4:** Effect of irrigation frequency on chlorophyll fluorescence parameters of mulch-free cotton.

Period	Process	F_0_	F_m_	F_V_	F_V_/F_m_	F_V_/F_0_	F_m_/F_0_	PIABS
Bud stage	P12	192.67 c	694.67 a	408.29 a	0.60 a	1.52 a	2.51 a	1.16 a
	P10	271.67 b	622.33 b	384.32 b	0.55 ab	1.49 a	2.24 b	0.84 b
	P8	245.00 b	622.00 b	335.08 c	0.52 bc	1.21 b	2.22 b	0.46 c
	P6	324.00 a	568.33 c	321.05 c	0.47 c	1.18 b	2.18 b	0.44 c
Blossom period	P12	289.33 b	916.33 a	682.54 a	0.70 a	2.51 a	3.50 a	3.52 a
	P10	278.33 b	852.00 ab	575.75 b	0.64 bc	1.88 b	3.39 a	2.79 bc
	P8	288.67 b	795.67 b	549.38 b	0.67 ab	2.36 a	2.71 b	3.2 ab
	P6	439.67 a	641.67 c	474.07 c	0.60 c	1.66 c	2.49 b	2.47 c
Boll stage	P12	288.33 b	993.33 a	750.93 a	0.80 a	3.91 a	4.91 a	9.51 a
	P10	243.33 b	990.00 a	711.43 b	0.74 b	3.28 b	4.06 b	9.12 a
	P8	386.00 a	920.00 b	745.26 ab	0.73 b	3.06 b	4.27 b	6.31 b
	P6	430.67 a	915.67 b	660.39 c	0.65 c	2.38 c	3.38 c	4.59 c
Lint stage	P12	193.33 b	861.67 a	406.19 a	0.60 a	1.53 a	2.53 a	2.29 a
	P10	189.00 b	630.67 c	381.63 ab	0.58 ab	1.33 b	2.33 b	1.48 b
	P8	219.67 b	679.00 b	365.02 bc	0.55 b	1.13 c	2.12 c	1.00 c
	P6	288.00 a	628.67 c	340.65 c	0.54 b	1.03 c	2.03 c	0.90 c

Note: The different lowercase letters in the same column indicate significant differences between treatments (*p* < 0.05).

## Data Availability

Data are available from the first author or the corresponding author on reasonable request.
